# Supplementary figures and images for: Dynamics and stability of directional jumps in the desert locust
Source: PeerJ. 2016 Sep 28;4:e2481. doi: 10.7717/peerj.2481 (PMC5045875; doi:10.7717/peerj.2481)

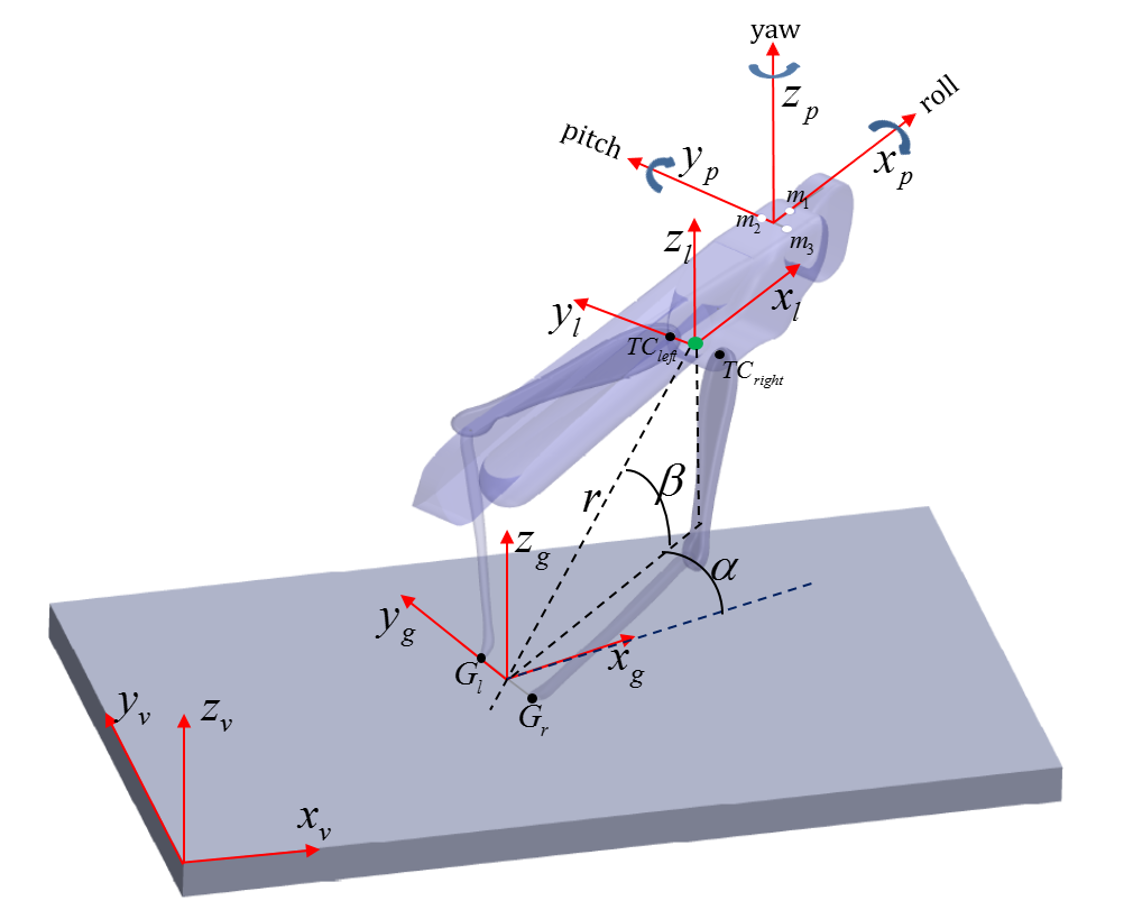

Supplement: Figure S1 — The positions of markers m1, m2, m3 obtained from video analysis are expressed in the video coordinate system (xv, yv, zv). The pronotum system (xp, yp, zp) is set according to the marker positions. The ground system (xg, yg, zg) is set according to the contact points of the hind legs with the ground (Gl, Gr). The locust system (xl, yl, zl) is parallel to the pronotum system and positioned between the TC joints (TCleft, TCright). During the jump, the locust position is the difference between the origins of the locust and ground systems expressed in spherical coordinates (α, β, r). [file peerj-04-2481-s001.png]

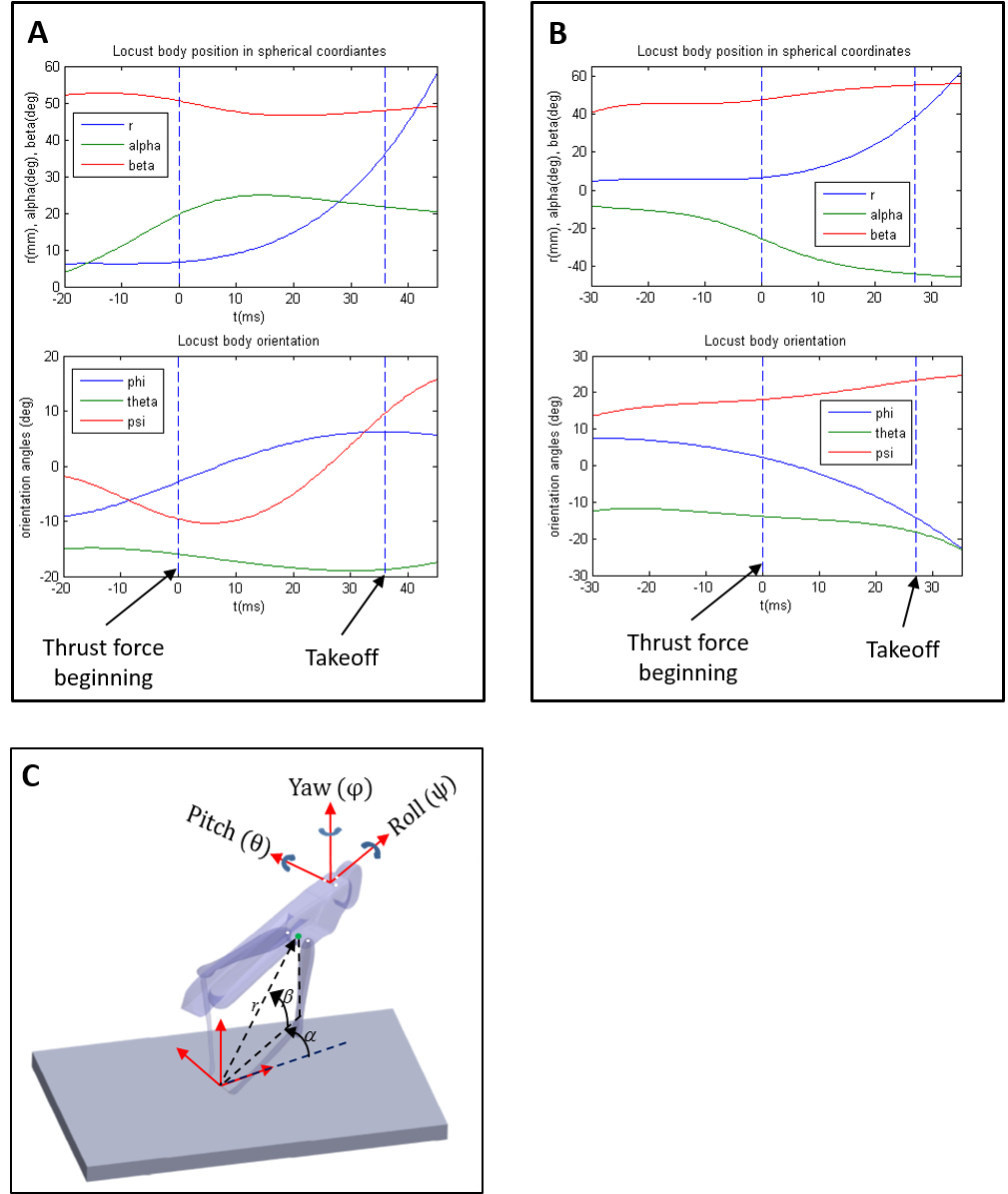

Supplement: Figure S2 — Each jump is represented by two graphs: the upper graph describes the position of the locust and the bottom graph describes the locust’s orientation. Two vertical dashed lines mark important events during the jump: The left line marks the moment when the hind legs start exerting thrust on the body. This moment was measured by noting the first frame in which the hind legs started to extend in each jump video. The right line marks the moment when the hind legs’ thrust force ends. This moment was measured by noting the frame in which the hind legs lost contact with the ground in each jump video. An image of the locust and the six coordinates describing its location and orientation are presented in figure S2C. A. First jump- The aiming manoeuvres begin about 20 milliseconds before the thrust force begins. During this phase the locust’s position changes only by changing the α angle between approximately 4 degrees at the beginning of the aiming manoeuver to approximately 20 degrees when the thrust starts. Meanwhile, during the same phase the locust changes its orientation: The pitch angle (θ) almost does not change; The yaw angle (φ) changes from approximately 9 degrees to the right to 0 degrees. The roll angle (ψ) changes from approximately 2 degrees to the left to 10 degrees to the left. Notice that the average velocity in which the roll was changed is approximately 400 degrees per second. In the second phase, the thrust phase, which takes place from the beginning of the thrust application till takeoff, the main change in the locust’s position is its propagation, which can be seen in the rise of r at approximately 30 mm till takeoff. During this phase also the orientation of the locust changes: The pitch and yaw angles (θ and ψ, respectively) continue changing in the same velocity till about 10 milliseconds before takeoff, when their velocity starts to reduce. The roll (ψ) on the other hand, changes direction and develops a higher velocity than in the previous phase. Th [file peerj-04-2481-s002.png]

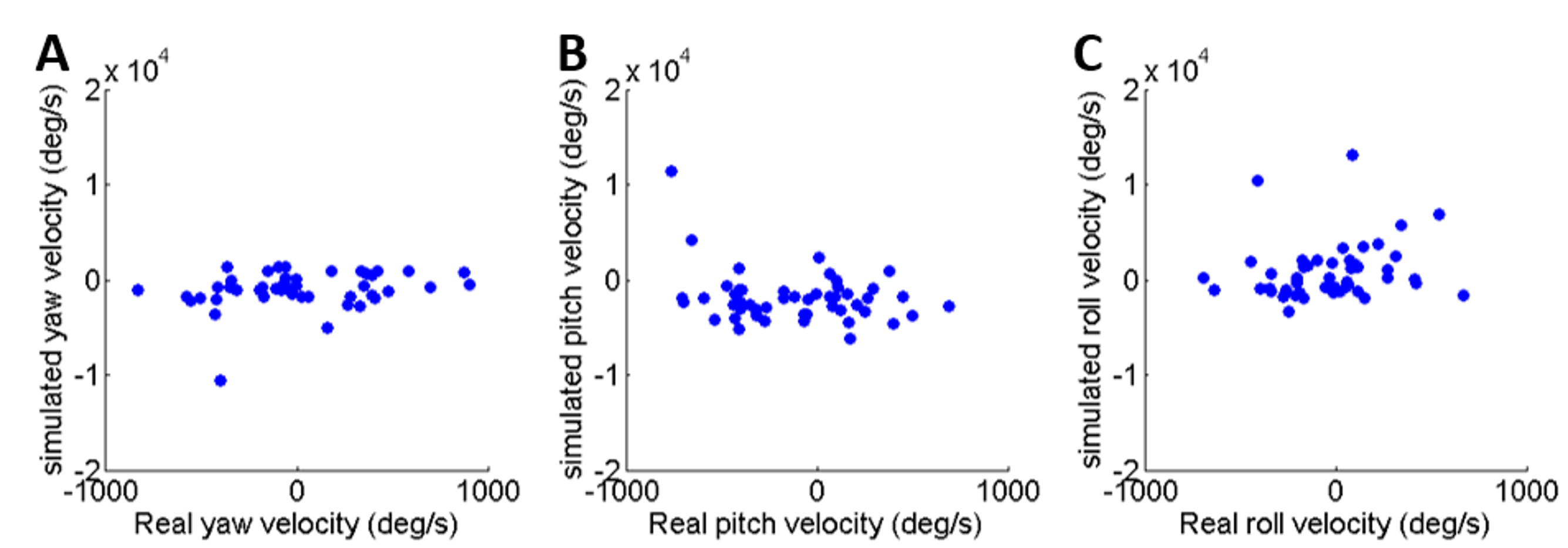

Supplement: Figure S3 — (A) Yaw velocity. (B) Pitch velocity. (C) Roll velocity. Please note that the x and y axes are not in the same scale. [file peerj-04-2481-s003.png]

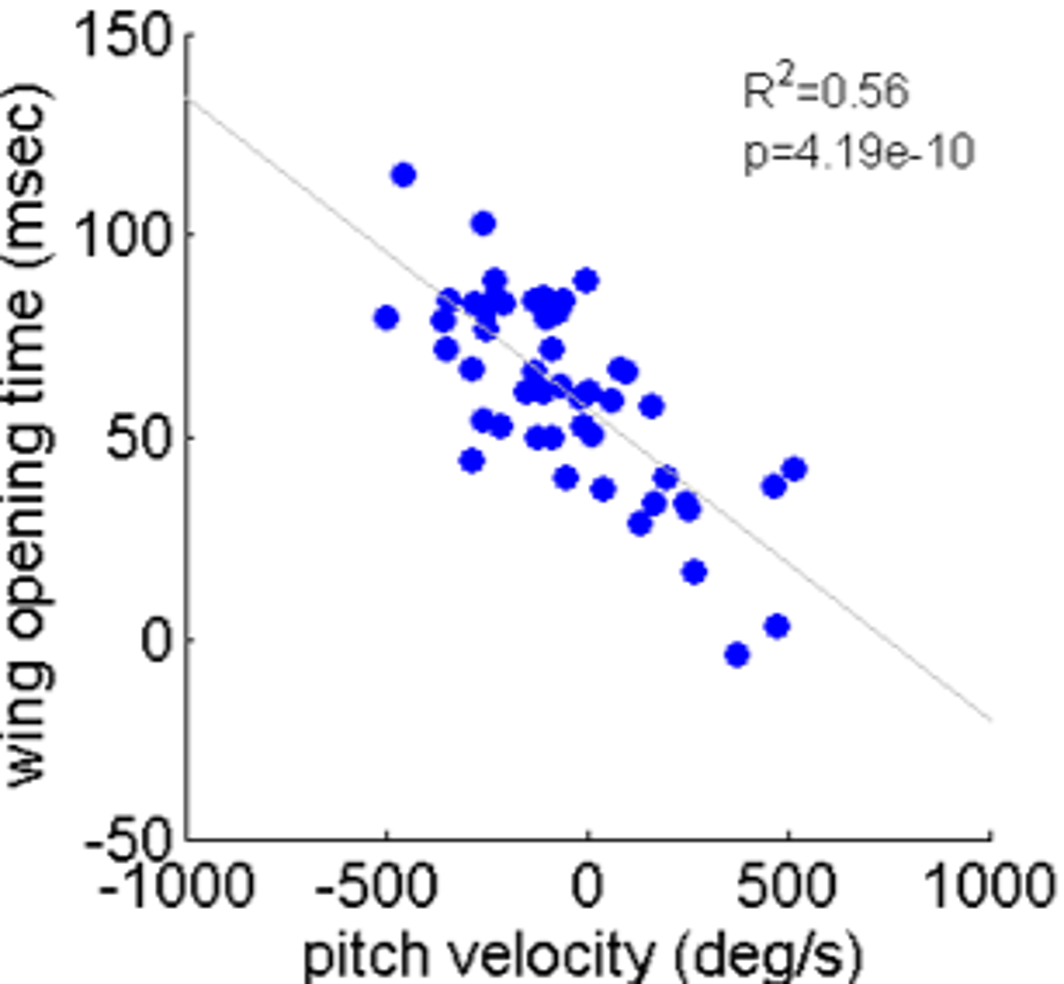

Supplement: Figure S4 — The lines denote linear regression (Analysis of variance of linear model, F-test) [file peerj-04-2481-s004.png]
