# Supplementary material for: Dynamics and stability of directional jumps in the desert locust
Source: PeerJ. 2016 Sep 28;4:e2481. doi: 10.7717/peerj.2481 (PMC5045875; doi:10.7717/peerj.2481)
Supplement: File S1 [file peerj-04-2481-s005.pdf]

Supplementary text

Coordinate systems and kinematic analysis. Four coordinate systems were defined in order to analyse data obtained from the video (Fig. S1): (1) the video coordinate system was attached to the jumping platform and the marker positions digitized from the videos are expressed in this system. (2) The ground coordinate system was attached to the locust's hind-leg contact points with the jumping platform. The $\hat{x}$ and $\hat{y}$ axes of this frame were established by a line connecting the hind-leg contact points and the $\hat{z}$ axis was set perpendicular to the jumping platform (Fig S1), its origin was set midway between the two contact points. Defining these two coordinate systems enabled us to differentiate between jumps in which the locust had rotated its whole body, including the hind-leg contact points, from jumps in which the body was rotated while the hind-leg contact points were kept stable (the latter was the common case in escape jumps). (3) The pronotum coordinate system was attached to the locust body. The origin of this frame was set midway between the two markers at the base of the pronotum. The xy plane was set to coincide with the dorsal surface of the pronotum. (4) The locust coordinate system was similar in orientation to the pronotum system but its origin was translated to midway between the TC joints. Establishing the origin and the axes of this frame in this manner simplified calculations and is common practice in robot modelling, where reference frame origins are set at joints, and axes are collinear with rotation axes. The jump trajectory is expressed as the translation and rotation of the locust system with respect to the ground system. Translation is described in spherical coordinates (Fig S1) and rotations are described using the intrinsic yaw-pitch-roll (YPR) rotation sequence convention.

After marker coordinates were obtained from the videos for each jump, the jump trajectory was reconstructed. The axes vectors of the ground coordinate system were computed:

$$\hat{y}_g = \frac{G_l - G_r}{\|G_l - G_r\|};\ \hat{z}_g = \hat{e}_3;\ \hat{x}_g = \hat{y}_g \times \hat{z}_g$$

Where: G_l and G_r are the left and right hind legs' ground contact points respectively.

The pronotum system's origin, axes were computed:

$$O_p = \frac{1}{2}(m_2 + m_3);\ \hat{x}_p = \frac{m_1 - O_p}{\|m_1 - O_p\|};\ \hat{y}_p = \frac{m_2 - m_3}{\|m_2 - m_3\|};\ \hat{z}_p = \hat{x}_p \times \hat{y}_p$$

Where: m_1 , m_2 and m_3 are the three markers drawn on the locust's pronotum.

Because the locust and the pronotum systems have the same orientation, their axes were in the same directions and their direction cosine matrices (DCM) with respect to the ground system

were equal. In each frame the instantaneous DCM (matrix R) was calculated (Diebel, 2006) and then used for transforming vectors from the ground to the locust system and the yaw, pitch and roll angles were also calculated through it. The digitized position of the TC joint was used to calculate the vector from the origin of the pronotum system to that of the locust system:

$$a_g = \mathrm{TC}_{left} - O_p;\ a_l = R \cdot a_g;$$

Assuming that the locust body is symmetrical with respect to a vertical plane coinciding with its longitudinal axis, the vector b_l connecting O_p to O_l was constructed by eliminating the second component of a_l . a_l was independently calculated in four different video frames and averaged to reduce error. The instantaneous position of the locust system could be found and described in spherical coordinates:

$$O_l = O_p - R^T \cdot b_l;\ \alpha = a\tan 2(O_l^y, O_l^x); \beta = a\tan 2(O_l^z, \sqrt{O_l^x + O_l^y});\ \mathrm{r} = \|O_l\|$$

Calculation of velocities was conducted by numerically differentiating the time varying position and Euler angles. To find the kinematics as experienced by the locust, the Euler angle time derivative were transformed to rotational velocities about the ground system axis, and then multiplied by the DCM to rotate to the locust system:

$$\omega_g = \begin{bmatrix} \dot{\psi}\cos(\theta)\cos(\phi) - \dot{\theta}\sin(\phi) \\ \dot{\psi}\cos(\theta)\sin(\phi) + \dot{\theta}\cos(\phi) \\ \dot{\phi} - \dot{\psi}\sin(\theta) \end{bmatrix};\ \omega_l = R \cdot \omega_g$$

References

Diebel J. 2006. Representing attitude: Euler angles, unit quaternions, and rotation vectors. Matrix 58:1516.

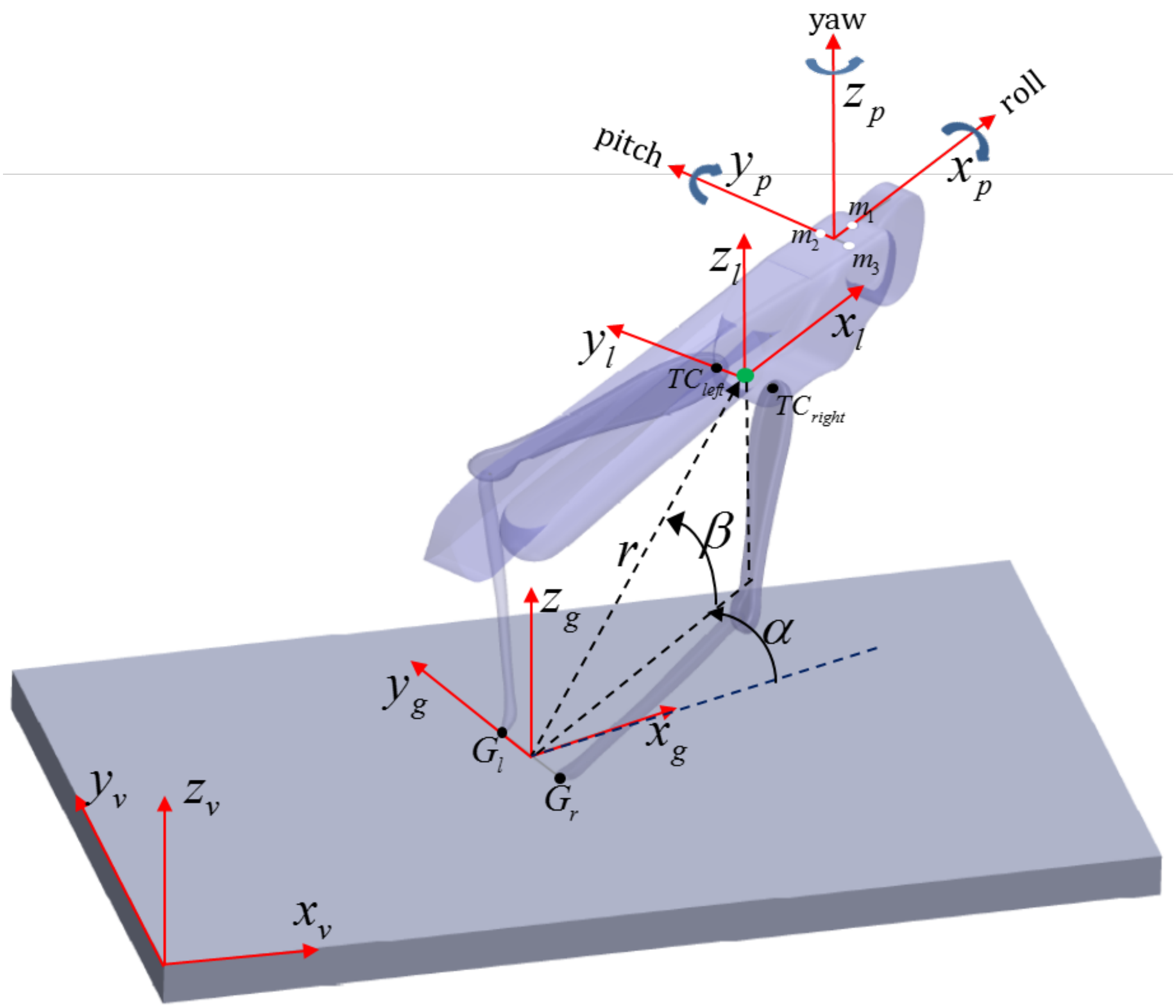

Fig. S1.

Definition of coordinate systems for kinematic analysis. The positions of markers m_1, m_2, m_3 obtained from video analysis are expressed in the video coordinate system (x_v, y_v, z_v). The pronotum system (x_p, y_p, z_p) is set according to the marker positions. The ground system (x_g, y_g, z_g) is set according to the contact points of the hind legs with the ground (G_l, G_r). The locust system (x_l, y_l, z_l) is parallel to the pronotum system and positioned between the TC joints (TC_{left}, TC_{right}). During the jump, the locust position is the difference between the origins of the locust and ground systems expressed in spherical coordinates (α, β, r).

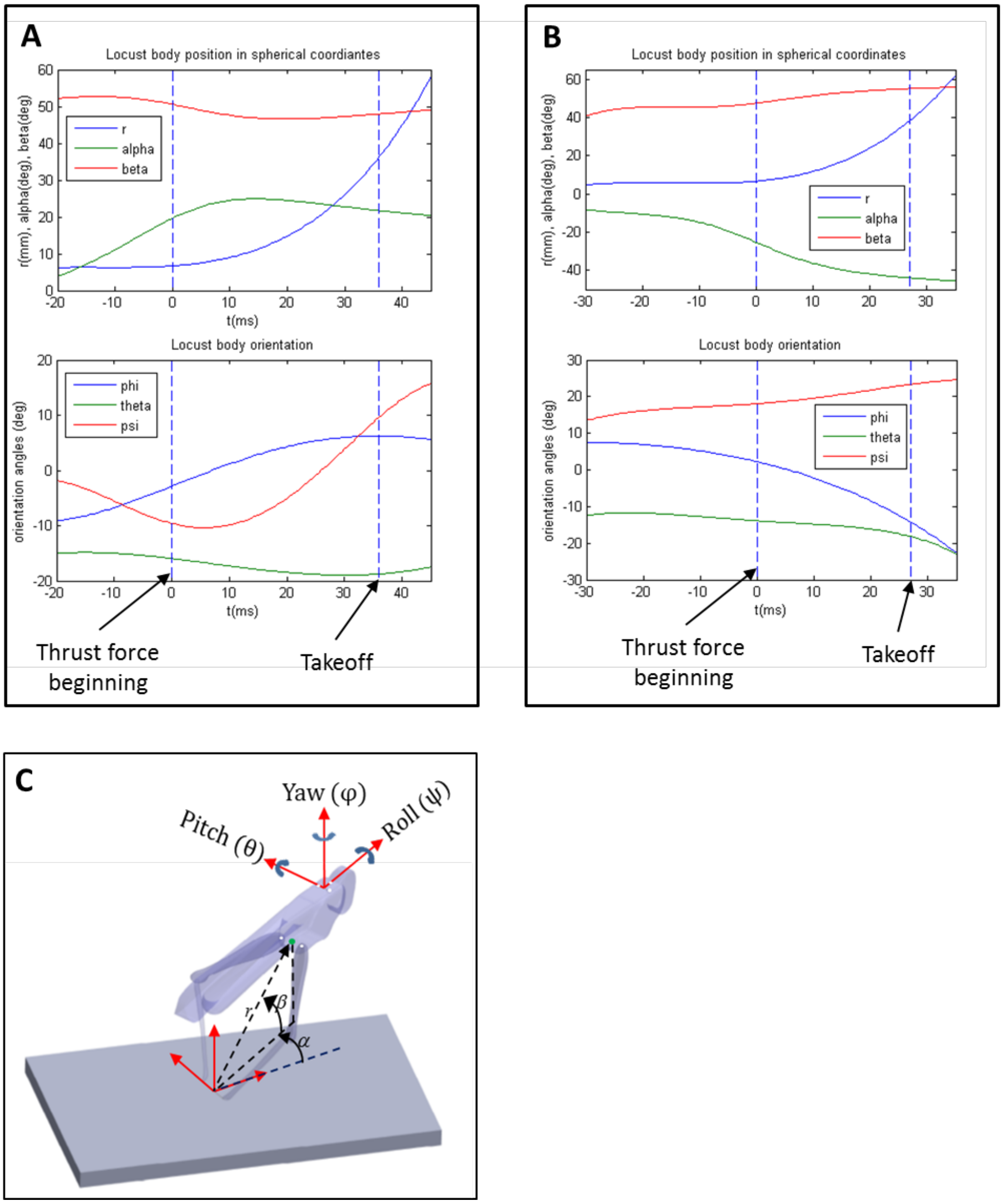

Fig. S2.

Time-course of two jumps from the beginning of the aiming manoeuvres till takeoff. Each jump is represented by two graphs: the upper graph describes the position of the locust and the bottom graph describes the locust's orientation. Two vertical dashed lines mark important events during the jump: The left line marks the moment when the hind legs start exerting thrust on the body. This moment was measured by noting the first frame in which the hind legs started to extend in each jump video. The right line marks the moment when the hind legs' thrust force

ends. This moment was measured by noting the frame in which the hind legs lost contact with the ground in each jump video. An image of the locust and the six coordinates describing its location and orientation are presented in figure S2.C for. A. First jump- The aiming manoeuvres begin about 20 milliseconds before the thrust force begins. During this phase the locust's position changes only by changing the α angle between approximately 4 degrees at the beginning of the aiming manoeuver to approximately 20 degrees when the thrust starts. Meanwhile, during the same phase the locust changes its orientation: The pitch angle (θ) almost does not change; The yaw angle (φ) changes from approximately 9 degrees to the right to 0 degrees. The roll angle (ψ) changes from approximately 2 degrees to the left to 10 degrees to the left. Notice that the average velocity in which the roll was changed is approximately 400 degrees per second. In the second phase, the thrust phase, which takes place from the beginning of the thrust application till takeoff, the main change in the locust's position is its propagation, which can be seen in the rise of r at approximately 30mm till takeoff. During this phase also the orientation of the locust changes: The pitch and yaw angles (θ and ψ, respectively) continue changing in the same velocity till about 10 milliseconds before takeoff, when threir velocity starts to reduce. The roll (ψ) on the other hand, changes direction and develops a higher velocity than in the previous phase. The change in roll in this phase is probably the result of torques produced by the thrust force. B. Additional jump time-course. C. Locust model with coordinate notations.

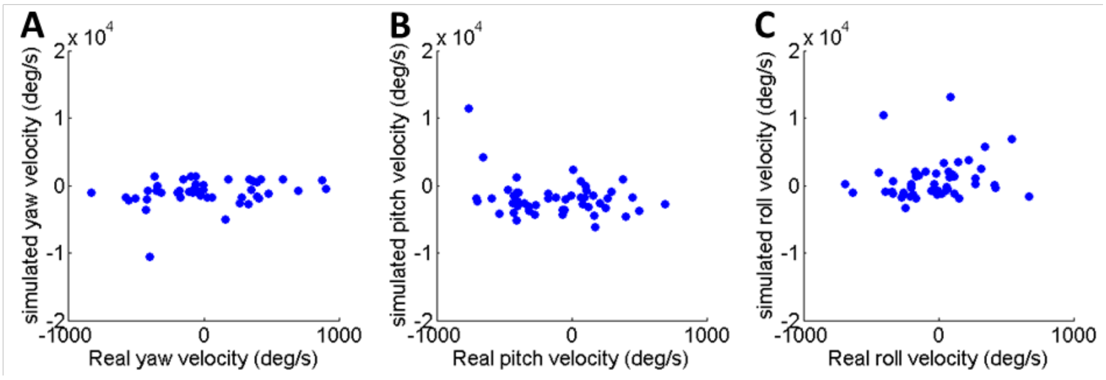

Fig. S3.

Comparison of rotational velocity at take-off between real and simulated jumps: A. Yaw velocity. B. Pitch velocity. C. Roll velocity. Please note that the x and y axes are not in the same scale.

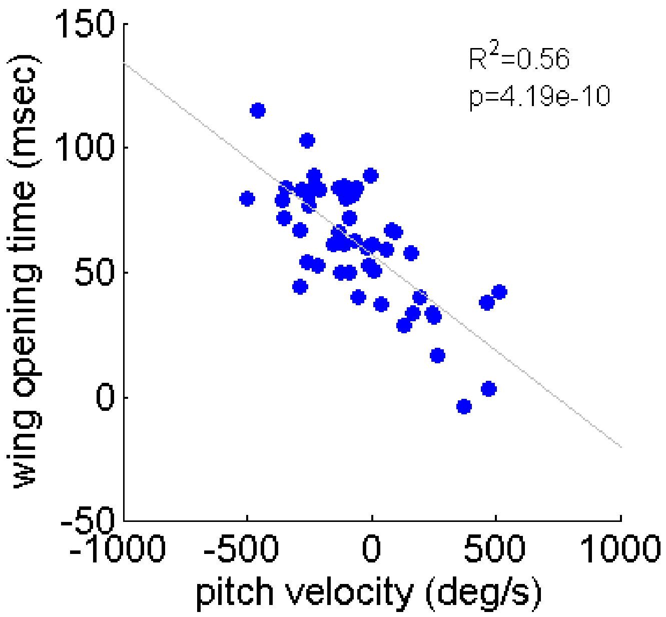

Fig. S4.

The timing of flight initiation as a function of pitch velocity measured 15 msec before take-off. The lines denote linear regression (Analysis of variance of linear model, *F*-test).
